# Technical Challenges for Smooth Interaction With Seniors With Dementia: Lessons From Humanitude™

**DOI:** 10.3389/frobt.2021.650906

**Published:** 2021-06-02

**Authors:** Hidenobu Sumioka, Masahiro Shiomi, Miwako Honda, Atsushi Nakazawa

**Affiliations:** ^1^ Advanced Telecommunications Research Institute International, Kyoto, Japan; ^2^ National Hospital Organization Tokyo Medical Center, Tokyo, Japan; ^3^ Graduate School of Informatics, Kyoto University, Kyoto, Japan

**Keywords:** Humanitude, dementia care, social robot, human-robot interaction, skill evaluation, dementia

## Abstract

Due to cognitive and socio-emotional decline and mental diseases, senior citizens, especially people with dementia (PwD), struggle to interact smoothly with their caregivers. Therefore, various care techniques have been proposed to develop good relationships with seniors. Among them, Humanitude is one promising technique that provides caregivers with useful interaction skills to improve their relationships with PwD, from four perspectives: face-to-face interaction, verbal communication, touch interaction, and helping care receivers stand up (physical interaction). Regardless of advances in elderly care techniques, since current social robots interact with seniors in the same manner as they do with younger adults, they lack several important functions. For example, Humanitude emphasizes the importance of interaction at a relatively intimate distance to facilitate communication with seniors. Unfortunately, few studies have developed an interaction model for clinical care communication. In this paper, we discuss the current challenges to develop a social robot that can smoothly interact with PwDs and overview the interaction skills used in Humanitude as well as the existing technologies.

## Introduction

Dementia is a leading cause of disability and dependency in senior citizens worldwide (Livingston et al., 2017). This syndrome not only weakens such cognitive functions as memory and reasoning but also induces various problems collected under the rubric of the behavioral and psychological symptoms of dementia (BPSD), such as agitation, aberrant motor behavior, anxiety, depression, apathy, delusions, and hallucinations. Although BPSD’s guidelines recommend that caregivers pay more attention to such people, strict adherence to such policies complicates caregiving and increases their burden and costs. Therefore, BPSD reduction is an important issue for caregivers who want to build good relationships with people with dementia (PwDs) and reduce their own burden (Selwood et al., 2007; Cerejeira et al., 2012).

Although pharmacological interventions can reduce BPSDs, non-pharmacological interventions are generally preferred as the first option to avoid adverse events and polypharmacy (Cerejeira et al., 2012). Among the many non-pharmacological interventions that have been proposed, researchers have identified the following consistent characteristics in the more effective ones: they are usually multimodal, provide individualized care, and train caregivers in skills that include optimizing communication with PwDs (Livingston et al., 2017).

Robotic technologies, which have mainly been introduced in elderly care to provide physical and mental support for seniors in multimodal individualized care, are usually used in rehabilitation and physical support. More recently, robot therapy is attracting attention as a form of mental support to reduce BPSDs in PwDs through interaction with pets or humanoid social robots (Broadbent et al., 2009). Previous studies identified various positive effects (Broekens et al., 2009; Robinson et al., 2013; Shibata and Coughlin, 2014; Pu et al., 2019), including improved psychological and physiological well-being (Mordoch et al., 2013; Costescu et al., 2014), better QoL (Chu et al., 2017), fewer problematic behaviors (Moyle et al., 2014; Jøranson et al., 2015), and less depression and anxiety (Petersen et al., 2016). Many of these studies use pet-like robots; such non-humanoid robots are preferred by seniors with mild dementia (Wu et al., 2012). But since PwDs and their caregivers want social robots that have verbal communication functions (Korchut et al., 2017), achieving social robots that satisfy the desire of PwDs for human interaction is essential. This idea is also supported by the fact that human interaction is the most enjoyable and long-lasting intervention for PwDs (Cohen-Mansfield et al., 2010). Therefore, creating social robots that can satisfactorily interact with humans is a major challenge in dementia care. However, it remains unclear how to design interactions with PwDs to reduce BPSD and what the technical challenges are.

Communication with PwDs is often difficult, resulting in caregivers who grudgingly provide treatment without much respect for their patients. Inappropriate communication is a main cause of BPSD (Cohen-Mansfield, 2000), and teaching caregivers how to effectively communicate with dementia patients ameliorates BPSDs (Selwood et al., 2007; Livingston et al., 2017). Improved communication skills among caregivers with their dementia patients can also reduce their own burdens (Adelman et al., 2014). This reduction is a critical factor in maintaining good relationships between seniors and their caregivers. Various approaches have been proposed for communication techniques to improve relationships with seniors to provide care based on personal dignity.

Humanitude^™^ is a multimodal method of comprehensive care that is attracting attention because it provides specific techniques for communicating with PwDs in actual care settings (Gineste and Pellissier, 2007; Honda et al., 2014). Humanitude consists of four care elements: three for communication, seeing, talking, and touching, and one that assists the physical act of standing. BPSDs and refusing care can be reduced in PwDs with this methodology (Biquand and Zittel, 2012; Simões et al., 2012; Ito et al., 2015; Honda et al., 2016; Melo et al., 2017b; Melo et al., 2017a; 2019; 2020; Figueiredo et al., 2018; Henriques et al., 2019; 2020). Humanitude provides systematic care techniques to communicate with PwDs in nursing care. If we analyze, model, and implement such techniques in social robots, we can create interactions that reduce such symptoms of PwDs. By analyzing Humanitude, we also believe we can clarify the differences between skilled and novice human caregivers and create a training tool for human caregivers. However, the scientific validation of its effectiveness is still in its infancy although Humanitude has been recognized for its effects and has already been implemented in many nursing homes. Therefore, there is limited information for researchers to understand Humanitude care. As a result, it is difficult to analyze, model, and implement Humanitude techniques into robots.

The purpose of this study is to summarize the key features of Humanitude’s communication skills and focus on the technical challenges that must be achieved with robots, based on books written by developers of Humanitude (Gineste and Pellissier, 2007) and the latest research by researchers who use it for care. We aim to develop social robots that have a good relationship with PwDs as well as evaluation systems that facilitate caregivers’ learning of communication skills in Humanitude. In the following, we first overview Humanitude and identify the key features of its care techniques. Then we summarize technical challenges to achieve social robot in which these techniques have been incorporated. Finally, we discuss other key evaluation challenges of the internal states of dementia patients and collaboration between human caregivers and the robots in Humanitude care.

## Humanitude^™^

Humanitude™ refers to a concept proposed in 1979 by Y. Gineste and R. Marescotti and the care techniques based on it. It emphasizes respect for the human dignity of the individuals who are receiving care and provides technical solutions for their caregivers and families. To date, Humanitude, which has been introduced in more than 600 hospitals and nursing homes in Europe, is beginning to carve out inroads in North America as well (Melo et al., 2017b). Several studies have shown significant reductions of aggressive behavior in patients (88.5%), less reliance on neuroleptics as well as cost effectiveness with a social return of investment (SROI: 4.07) (Ito et al., 2015; Pimentel and Martins, 2015; Honda et al., 2016).

**FIGURE 1:**
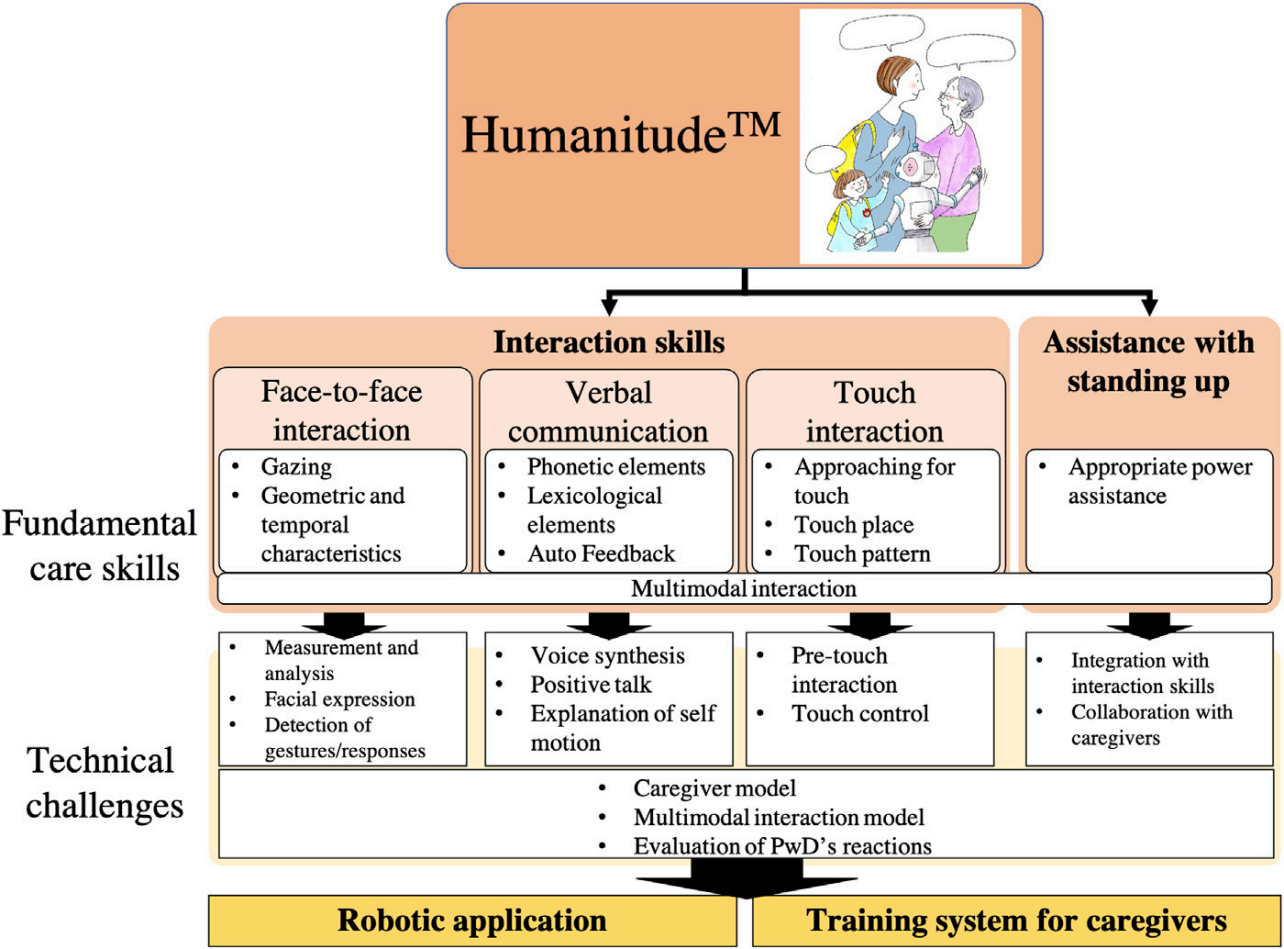
Fundamental skills in Humanitude and technical challenges.

Humanitude emphasizes the development of good relationships between caregivers and care receivers. The following are its three key communication elements: seeing (face-to-face interaction), talking (verbal communication), and touching (touch interaction) (Figure 1). For humans, maintaining a standing posture is important for spatial recognition, positive physiological effects, and individual self-consciousness (Lopez et al., 2008; Lopez and Blanke, 2010; Wilson et al., 2013). For this reason, the fourth element, assistance with standing up, is also crucial for care. Caregivers are directed to assess the stage of care needed by each PwD and assist him/her to stand and walk on his/her own as much as possible during care. In the following, we discuss these care elements in detail.

### Face-to-Face Interaction

When we fail to obtain enough information about our environment due to a decline of sensory modalities, we tend to feel anxious and passive (Gineste and Pellissier, 2007). Therefore, the characteristics of sensory processing must be understood in seniors to communicate with them. In particular, face-to-face interaction plays an important role in initiating communication and conveying the impressions of another person. Humanitude facilitates communication and good relationships with PwDs by emphasizing three aspects in face-to-face interaction: eye contact, its geometric characteristics, and its duration.

#### Gazing (Eye Contact)

Gazing is a core element in the Humanitude skill system, which is mainly related to the following eye contact (mutual gaze) states: 1) extending eye contact, 2) maintaining it during communication, and 3) reducing durations without it. The objective of these skills is to help PwDs continue to pay attention to caregivers since the former are easily distracted by other stimuli, such as other individuals or objects. In Humanitude, these efforts to preserve continuous communication can lead to smoother interaction with PwDs.

In psychology, eye contact is the most basic state that indicates a readiness for communication with another person (Senju and Johnson, 2009). Doctors who exhibit patient-centered behavior make more eye contact with their patients during consultations than those who do not (Gorawara-Bhat and Cook, 2011). Caregivers must make eye contact with PwDs when they start caring for them and maintain it during their care. Establishing eye contact with another person in a social relationship also facilitates the memory of that person’s face (Conty, 2016; Lopis and Conty, 2019) and shared conversation (Fullwood and Doherty-Sneddon, 2006). Eye contact activates conversations with PwDs and supports their memories (Lopis et al., 2019). As the baseline, older people have difficulty with contrast vision and reduced binocular summation (Gillespie-Gallery et al., 2013), and required four times longer time than youger participants to recognize the target in the functional visual filed test (Coeckelbergh et al., 2004). Addition to these normal physiological change, visual sensitivity of PwDs is reduced throughout the visual field (Trick 1995). Therefore, Humanitude suggests that caregivers voluntarily engage in eye contact with them.

#### Geometric and Temporal Characteristics in Face-to-Face Interaction

Humanitude points out the importance of geometric properties when caregivers make eye contact with PwDs. The impression perceived by PwDs depends on how the eye contact is made. When eye contact is established from the front and at the same level, it creates a positive impression that conveys security, trust, and friendship. Eye contact from the side of the face without directly looking at the patient or from above creates a negative impression, such as anxiety, frustration, and anger. Physicians who behave in a patient-friendly manner often make eye contact at the same level as the patient (Gorawara-Bhat and Cook, 2011). Humanitude emphasizes the importance of such eye contact with patients from the front and at the same level or under.

Humanitude also discusses the distance between caregivers and PwDs during eye contact. Close proximity communicates intimacy and trust in PwDs. It also draws attention to the caregivers of a PwD who has poor visual and attentional conditions. Humanitude recommends an eye contact distance that is closer than the distance generally observed in adult communication: around 20–30 cm, which is dependent on the cognition level of the care receivers. Caregivers should approach PwDs from the front because this tactic is a reasonable way to display their face to patients, based on such findings as face recognition in Alzheimer’s disease, which is the most common type of dementia (Adduri and Marotta, 2009).

Humanitude also defines eye contact duration. Caregivers should maintain it for longer than 0.5 s so that PwDs become aware that they are being watched. Although prolonged eye contact is recommended, silence can also become uncomfortably eerie to seniors. Caregivers should respond by talking to a PwD within 2 s after establishing eye contact (Honda et al., 2014).

### Verbal Communication

The second communicative element is verbal communication, which has two categories. One is such phonetic elements as tone, speed, and volume; the other is lexicological elements: vocabulary. In care settings, all verbal communication is conveyed with non-verbal information. Generally, most of the topics focused on by caregivers concentrate on their work, and their conversations are brief. In fact, a report described that verbal communication lasted only 2 min each day for a bedridden dementia patient in a long-term care facility (Gineste and Pellissier, 2007; Honda et al., 2014). Caregivers often become discouraged by laconic PwDs who fail to respond or show irrelevant answers. Humanitude argues that good communication skills must be developed based on both phonetic and lexicological elements as well as a technique called Auto Feedback where caregivers talk continuously even when care receivers respond inadequately.

#### Phonetic Elements: Tone, Speed, and Volume

Phonetic information includes tone, speed, and volume. PwDs have disproportionate atrophy in the medial, basal, and lateral temporal lobes, and medial parietal cortex, which lead difficulty in understanding the meaning of linguistic information (McKhann et al., 2011). However, their amygdala essentially maintains its function and analyzes emotional meaning of paralinguistic information. Hearing loss is the highest population attributable factor for dementia (Livingston et al., 2017, 2020). Due to this, people taking care of PwDs have a tendency to speak loud and high pitch voice to communicate. These tones convey negative emotional prosody to PwDs. To avoid this condition, United Kingdom National Health System^1^ recommended to calm, slow, gentle and low voice, which is also recommended in Humanitude.

#### Lexicological Elements: Positive Words

Another category of verbal communication is lexicological elements. Vocabulary is critical to convey positive information. The words used by caregivers generally communicate such requests as “Please open your mouth” or “Hold still” or apologies like “I’m sorry that you are in pain by my procedure.” To establish good relationships with PwDs, selecting positive words is key. There are other expressions to deliver the same communication and care in different words, conveying identical meaning by adding a positive emphasis: “Thank you for keeping your mouth open, that is a big help.”

During and after the caregiving process, the caregiver must continue to give positive feedback to seniors about the care’s content and their reactions. For example, after the caregiver get a PwD changed her clothes, a positive explanation can connect this action to a good feeling: “New pajamas makes you feel so comfortable, don’t they?” “I like to see you smiling at me.”

#### Auto Feedback

Maintaining verbal communication is difficult when the other person fails to respond. Type of silence between patients and healthcare professionals falls into three categories; awkward, invitational and compassionate (Back et al., 2009). Verbal communication failure with PwDs belongs to awkward silence. Sensory deprivation speeds up the degenerative changes normally associated with aging and enhances the loss of functional cells in the central nervous system (Oster, 1976) and deteriorate dementia (Humes and Young, 2016). To minimize the sensory deprivation, verbal information should be maximized to PwD. Even with no response from PwDs, a caregiver must keep talking with positive language to avoid silence. Proposed by Humanitude, “Auto Feedback” is a skill for maintaining verbal communication during caregiving that describes caregiving actions. By talking about oneself, the caregiver can present verbal stimuli to the PwD without depending on her/his responses, creating a chance to elicit a response. In addition, by explaining their own actions during care, caregivers can increase the feelings of security sensed by PwDs about their care.

Auto Feedback consists of three phases. Caregivers first ask the PwDs to behave voluntarily since Humanitude’s basic element is to encourage them to move as much as possible by themselves. If the person does not respond to the word of caregivers, next, caregivers tell care receivers what they are going to do. For example, the caregivers must clearly describe to their patients what exactly they are going to do before actually doing it: “I’m going to wash your arms now.” Then they present explanatory information: what is being done. For example, “Now I’m raising your left arm.” Although mastering such a concise and clear communication method appears quite simple, caregivers require an average of one to three months of training (Gineste and Pellissier, 2007). This phase of Auto Feedback can increase verbal communication during daily care by four to six times, which can prevent sensory deprivation of PwDs.

### Touch Interaction

Touching PwDs by caregivers is another common behavior in various daily support activities, such as changing clothes, bathing, and feeding. Touching plays an important role in communication with them in the mechanism of activating c-tactile afferents to excrete oxytocin (Walker et al., 2017). In Humanitude, there are three touch categories. The first is a validating touch (pleasant touch) that builds and maintains good relationships with others, such as hugs and handshakes. The second is an aggressive touch (unpleasant touch), such as hitting or pulling, which causes pain. The last type is a necessary touch (unpleasant but acceptable in the context), which is often uncomfortable but required during medical examinations or dental treatments.

Touch interaction is another opportunity for communication to develop good relationships. A person brings positive or negative meaning to another, depending on the kind of touch or how it is performed (Hertenstein et al., 2009). Since touching behavior may infringe on a person’s privacy, e.g., changing diapers or potentially invasive medical procedures, a friendly attitude and an intimate relationship must be maintained before and during such touching. For this reason, Humanitude determines the techniques, how to touch PwDs in the care.

#### Approaching for Touch

*Aggressive touches* are never acceptable. Due to their cognition decline, PwDs has difficulties to understand *neccessary touches*. Caregivers must consider what message is being conveyed by their touch. *Aggressive touches* must be avoided. *Necessary touch* must be made as comfortable as possible. For example, to avoid conveying negative information, caregivers should not approach the arm of a PwD with grabbing from above. They should approach to their arms to support from below.

#### Touch Place

The place that is touched is important in Humanitude. Tactile stimuli are received by the brain’s somatosensory cortex. As Penfield describes, the size of the receptive area depends on the body part. The area corresponding to the hands, face, and mouth is large; the area corresponding to the legs and arms is small (Penfield and Boldrey, 1937). Even if we touch a person in the same way, the effect on his/her brain will be different depending on which part was touched. Therefore, to avoid startling an PwD by suddenly receiving too much information, caregivers should first touch the parts that convey less information, which is the upper arms, shoulders, and back. Sensitive areas should only be touched when absolutely necessary: the hands, the face, and the genital region.

#### Touch Pattern

Like caressing a baby or loved one, a slow, gentle touch over a large area is fundamental in Humanitude, which is to activate c-tactile neurons (Walker et al., 2017). Such an approach to touching is necessary so that PwDs have positive impressions about caregivers. Humanitude recommends to touch with the fingertips at first, and followd by the palm, like landing an airplane. If a touch is too light, it might connote an unwelcome sexual implication or an awkward reluctance to touch; a certain amount of force should be applied. In Humanitude, the applied force during a touch should fall within a certain range. It is also recommended that one of the hands should always touch the PwD during care to keep conveying a positive relationship. When the touch is finished, the hands should leave the body in the opposite order at which the touch was started like an airplane that is taking off.

### Assistance With Standing up

Human has ability to stand up and walk. The harmful effect of prolonged bed rest has been pointed out (Allen et al., 1999) and healthy older adults showed significant functional decline by 10 days bed rest (Kortebein et al., 2008), The amount of information from peripheral receptors about position and perception is more in upright than supine (Lopez and Blanke, 2010). Also, among the patients in altered mental status showed significantly more arousal and awareness in upright position than bed ridden. The main goal of Humanitude is to maintain the health of seniors and allow them to live a life with dignity by helping them stand and walk, and accumurate the duration of standing up 20 min per day to prevent being bed ridden. For this purpose, daily care is important opportunities for maintaining the health since caregivers can assist seniors with standing and walking in the care. In fact, Humanitude offers many techniques that provide walking assistance. The key is letting seniors stand and walk by themselves to maximize their muscle strength.

During the standing up and walking assistance, the caregiver should always use more than two out of three modes of communication elements; visual, verbal, and tactile interactions to present consistent and positive stimuli through at least two or more visual, verbal, or tactile interactions. Consistent presentation of positive stimuli among multiple sensory inputs is crucial. Since PwDs often suffer cognition and perception declines, information that is presented in a single modality may be insufficient. Information must be presented in a multimodal manner to facilitate the transmission of positive information to seniors.

Multitasking ability is declined with age and especially in PwDs (Clapp et al., 2011). When more than two caregivers talk to a PwD in the care, simultaneously, the person becomes confused due to overwhelmed information. Therefore, in Humanitude, the roles must be divided so that the stimulus of each modality comes from the same person. For example, in bathing care, caregiver A is in charge of face-to-face interaction and verbal communication to draw attention of care receiver, while caregiver B is washing the body using the technique of touch without saying words. This strategy of Humanitude is called “the master and the hidden player.”

### Care Procedure to Build Intimate Relationships With Seniors With Dementia

**FIGURE 2:**
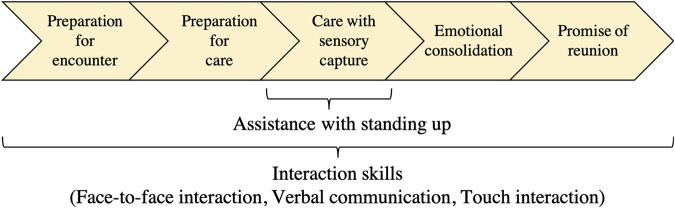
Five consecutive stages in Humanitude and related skills.

To make care more acceptable, Humanitude provides all of its care with one sequence that consists of five stages: 1) preparation for an encounter, 2) preparation for care, 3) care with sensory capture (provide care with multimodal approach), 4) emotional consolidation, and 5) a promise of reunion. In all the stages, the interaction techniques play an important role to build good relationships with PwDs and smoothly conduct care, while the assistive techniques for standing up during the care is worth for somatosensory input and to maintain muscle strength (Figure 2). First, during preparation for encounters, caregivers make the PwDs aware of their own presence. After they become more aware, the caregiver prepares for the care by multimodal communication to reach an agreement on care at the second stage. The key point in the first and second stages is that the caregiver should give the PwD the impression that he/she has come to see them not as a work responsibility but to spend good time together. The third stage provides the actual care for them by presenting positive stimuli with multimodal communication to make them realize that the care is a good experience. When the third stage is completed, the fourth and fifth stages build positive impressions of the care in the memory of the dementia patients and express that the caregiver also enjoyed spending the time with them. Since emotional memories stay longer even for PwDs, adequately providing stages four and five increases the likelihood that the next care will be more readily accepted.

Humanitude also emphasizes the importance of the caregiver’s contingent responses in interacting with PwD through all states. The adaptive/contingent interactions that the caregiver pays attention to the PwD’s behavior and responds contingently is one of the keys to realized good care interaction with PwDs, especially those who are non-verbal. Recent studies about intervention to PwD show that the adaptive interaction that uses non-verbal modalities for communication has the potential for promoting and supporting communication between people living with dementia who cannot speak and those who care for them (Ellis and Astell, 2017).

## Technical Challenges Toward Artificial Systems That Incorporate Humanitude Techniques

We have identified and described the basic elements of the care techniques proposed in Humanitude. Caregivers usually acquire them by special training. If we analyze and model these techniques, we can develop social robots that provide more elderly centered support as well as a supportive system that helps human caregivers acquire them.

To implement Humanitude techniques in social robots, researchers can address the development of three types of robotic systems: a robot as a caring teacher, a robot as a second caregiver in a team with a human, and a robot as a primary caregiver. As a first step, robots would work as a caring teacher to improve human caregivers’ abilities by sensing their actions and providing feedback based on modeled knowledge of Humanitude techniques. The reason is that current robot systems would not have enough physical support capabilities in caregiving, instead of rich sensing abilities to observe people’s behaviors. Next, robots would behave as a second caregiver, supporting human caregivers through physical collaborations by following advances in their hardware capability. After increasing the physical capabilities of robots, they will work as main caregivers, but it is still far from current situations. Based on these considerations, modeling of Humanitude technique will provide essential knowledge for robots to behave as care teachers firstly.

However, it remains unclear to what extent current technologies can satisfy the techniques and what technologies should be developed to implement Humanitude-based care into artificial systems. In this section, we discuss the technical issues that must be solved to achieve Humanitude care techniques and know-how in artificial systems.

### Technical Challenges for Face-to-Face Interaction Analysis

#### Measurement of Face-to-Face Interactions

For the purpose of human communication analysis [e.g., detection of Autism Spectrum Disorder (ASD) or social interactions], several computer-vision-based methods have been developed for detecting eye contacts (mutual-gaze) (Marin-Jimenez et al., 2019) or joint attention (Recasens et al., 2015). These method uses third-person-video where a filmer took a video.

Another approach is the use of first-person videos which are taken from the viewpoint of a caregiver from a frontal direction using a head-mounted wearable camera (first-person camera). For a social robot, first-person videos can be captured by a camera embedded in the eye-pupil or the forehead. From these videos, face-to-face postures (distances or angles) or eye contact states can be obtained using facial detection and/or machine learning techniques (Chong et al., 2020; Mitsuzumi et al., 2017). Similar systems such as wearable eye trackers (Chong et al., 2017) or proximity sensors (Hachisu et al., 2018) can be used for face-to-face interaction analysis as well. One advantage of a first-person camera is that it requires no third-person filmer. This is quite important for communication analysis with PwDs.

#### Analysis of Face-to-Face Interaction

Finding essential elements in face-to-face communication with PwDs is vital for smooth interaction for both caregivers and social robots. Thus, a number of studies have analyzed conversations with PwDs. O’Brien et al. described the conversational analysis approach for evaluating the skills of care learners before and after taking care-training courses. They compared the occurrence of communication techniques before/after training through video analysis and showed their effectiveness (O’Brien et al., 2018). Ishikawa et al. developed an analysis and training system for interaction with PwDs where an annotator rated the quality of the skill elements (gaze, speech, touch, and comprehensibility) for a conversational video. Trainees learn current interaction behavior through the system’s visualized feedback (Ishikawa et al., 2016).

Little work has been conducted on automated analysis for PwD interactions. Nakazawa et al. used a first-person video from the caregiver’s view while communicating with a PwD (Nakazawa et al., 2020). The relative facial posture (distance and angles) is automatically detected by the video and distinguishes skill levels using statistical analysis. They identified behavioral differences among care experts, middle levels, and novices. The expert and middle care levels look at the face of the care receivers more frequently; the face-to-face distances and rotations are also different among these three groups.

#### Facial Expression of PwD and Caregivers

Since facial expressions are a key role in PwD interactions, the automated recognition of them is a vital technique for evaluating levels of care skills and communication robots that have true interactivity. Facial expression analysis has two roles.

One is the facial expression analysis of care receivers/PwDs for sensing their status/mood. However, existing facial-expression recognition algorithms are mainly tuned for younger adults and posed (non-natural) conditions (Ebner et al., 2010). Therefore, few reliable techniques are applicable for recognizing the spontaneous facial expressions of PwDs. Collecting and annotating the facial expression dataset of PwDs is an important task to encourage studies. The second role is obtaining the facial expressions of caregivers as they interact with PwDs. Although much research literature has described the importance of positive facial expressions while communicating with PwDs, the relations remain unknown between the existing/magnitude of the positive/negative facial expressions of caregivers and the reactions of care receivers. Further studies must explore the effects of caregiver’s facial expressions for smooth communication with PwDs.

#### Detection of Facial Gestures/Responses of PwDs

Since PwDs suffer from impaired motor skills, the amplitudes of their gestures or facial expressions are quite small. Therefore, distinguishing between purposive and non-purposive movements is quite difficult, even by human caregivers. Moreover, they sometimes express their responses in such non-typical ways as blinking. Thus, novice caregivers often overlook these signals, a situation that leads to miss-communication with PwDs. Automated detection for such nuanced PwD responses is crucial and challenging in AI and Robotics. We have identified the following three key technical points of these tasks: 1) construction of well-annotated datasets, including videos from multiple viewpoints, 2) developing algorithms to detect slight movements that can discriminate between true signals and non-purposive behavior, and 3) temporal interaction analysis that takes the actions of caregivers into account for recognition.

### Technical Challenges for Verbal Communication

One of the crucial challenges for verbal communication is voice synthesis that makes PwDs feel comfortable since Humanitude suggests that it is essential for a robot to speak in a calm, slow, gentle, and low voice to make PwDs feel comfortable. There have been various efforts to synthesize emotional speech (Schröder, 2001). Some commercial software is already capable of synthesizing emotional speech which expresses some positive emotion such as happy and calm. However, it is unclear whether these synthesized expressions are helpful to make PwDs feel comfortable. It is important to verify the effect of the existing emotional speech on PwDs. Another issue is to synthesize emotional speech based on the speech of caregivers who learned Humanitude. To achieve this, we need to collect their speech during the interaction with PwDs and build a “Humanitude speech” corpus. Such a corpus is helpful for us to make speech synthesis systems for care robots by using machine learning methods such as hidden Markov model (Yamagishi et al., 2005; Nose and Kobayashi, 2013; Lorenzo-Trueba et al., 2015) and deep neural network (DNN) (Lorenzo-Trueba et al., 2018).

So that seniors positively experience their care, the robot also needs to describe the appropriate positive evaluations for each element of care. A database of care situations and their corresponding positive comments is required. Honda et al. developed a system that analyzes the multimodal behavior of Humanitude experts while performing such care (Ishikawa et al., 2016; Honda et al., 2017). Such a system helps model positive utterances based on specific situations.

To achieve Auto Feedback, which is one main technique for verbal communication in Humanitude, the robot needs to explain its actions. The simplest implementation of this function is preparing predefined scripts for each task in advance and playing them back since daily care tasks are determined to some extent. This approach is especially effective for informing the PwDs about what the caregivers are going to do. But such an implementation is not adaptive for accidents during care. Another approach could implement in a robot the ability to explain its own actions. Little research has investigated the possibility of learning the relationship between a robot’s actions and their corresponding explanations (Taniguchi et al., 2019). Platter et al. proposed bidirectional mapping between the whole-body motion of a humanoid robot and language using deep recurrent networks (Plappert et al., 2018). Yamada et al. proposed paired recurrent autoencoders that bidirectionally translate a robot’s actions and language (Yamada et al., 2018). In these studies, although the robot’s joint information is used as sensory input to the networks, the robot is expected to explain the caregiver’s behavior and provide Auto Feedback by combining their networks with recent methods that estimate human posture from camera images, such as OpenPose (Cao et al., 2021).

In addition to these challenges for verbal communication in Humanitude, robots must also require the basic abilities to chat with PwDs to improve their communication. A robot needs to recognize questions, replies, and other statements from them. Since the speech features of PwDs are different from the general population (Toth et al., 2018), for the speech recognition for PwDs, a speech recognition system must be specifically created for them (Russo et al., 2019).

### Technical Challenges for Touch Interaction

Due to the recent development of various robotic hands and machine learning approaches, social robots will be able to physically and safely touch people in the near future. However, social robots need to consider what kinds of touch behaviors would be acceptable by people because such considerations are different aspects from safe and physical touch. In fact, caregivers need to consider various factors for acceptable touches from patients, as described in section *Touch Interaction*. To enable Humanitude techniques by social robots, similar factors must be addressed. This section summarizes several essential factors for acceptable touch interactions from robots to people in the Humanitude context.

Firstly, we focused on a situation where people are approaching for touch, i.e., before-touch situation. Based on the described manners and considerations of section *Approaching for Touch*, we investigated related works that focused on before-touch interaction between people and robots. Based on these past studies, we thought that social robots should consider pre-touch situations for natural and acceptable touch interactions, similar to conversational interactions. For example, before talking with another person, people adjust their position relationships based on personal space (Hall, 1966). Many social robots also consider such position relationships before interacting with people as pre-conversational interactions (Obaid et al., 2016; Li et al., 2019; Saunderson and Nejat, 2019). Following such concepts, robotics researchers investigated the pre-touch reaction distance around a face between people and reported that the average distance is about 20 cm (Shiomi et al., 2018). They developed an android robot that reacted to the touch behaviors of people and concluded that a 20-cm threshold as a pre-touch reaction distance is more natural and human-like than personal space as a pre-touch reaction distance. This knowledge is useful for designing robot’s approaching behaviors before touch-based care.

In the before-touch situation, as described in sections *Face-to-Face Interaction and Technical Challenges for Face-to-Face Interaction Analysis*, gaze behaviors are also important in touch contexts because related works reported that a robot’s gaze in touch interaction implies its intention and changed perceived impressions of touched people. For example, Hirano et al. investigated the effects of gaze behaviors and touched timing when a robot touches participants (Hirano et al., 2018). They reported that participants preferred a gaze behavior that only looks at their faces during a touch more than a gaze behavior that looks at their faces, hands and returns to their face. Although this study did not investigate the duration effects of looking at faces, its results suggest the importance of face-to-face interaction as promulgated in Humanitude. They also investigated the effects of the robot’s gaze height and speech timing for touching in a nursing context (Shiomi et al., 2020a). Before-touch speech timing was preferred to after-touch speech timing, although the gaze height did not significantly improve robot-initiated touches’ feelings. Interestingly, this speech timing result contradicted a phenomenon from a past study that concluded that after-touch timing was preferred by participants in robot-initiated touch situations in a nursing context (Chen et al., 2014). Although different results described speech timing in touch situations, we believe that before-touch speech timing is better for a Humanitude context because after-touch speech timing might convey negative impressions to patients (Gineste and Pellissier, 2007; Honda et al., 2014).

We described the importance of considering touch place and patterns in the context of Humanitude in sections *Touch Place and Touch Pattern*. In fact, several human-human and human-robot touch interaction studies investigated the effects of touch place and patterns in influencing the perceived emotions, intimacy, and comfortableness. For example, human science literature has investigated the effects of touching speed on the impressions of comfort and reported that a rate of 5 cm/s was evaluated more positively than 0.5 or 50 cm/s (Essick et al., 1999). This knowledge is widely used in the human-robot interaction research field (Shiomi et al., 2017a). Another study developed a human-imitation hand to investigate the subjective and physiological effects of gentle stroking motions by a robot and reported that stroke speed and rate positively affect (Ishikura et al., 2020). To create comfortable touches from robots, several researchers have focused on the effects of warmth. For example, Block et al. developed a robot that provided a warm hug using chemical warming packs and reported that the warmth improved its hugs’ perceived impressions (Block and Kuchenbecker, 2019). Another study reported that a robot’s skin temperature influences users’ perceptions and evaluations (Park and Lee, 2014). These studies provided rich knowledge about robots’ body temperature design, particularly those that need to touch patients.

Touch place and patterns also influence conveying emotions, which is essential for social robots to interact with PwDs smoothly. Based on past related studies about touch place and patterns in human-robot touch interaction, Robotics researchers have already focused on this topic, i.e., conveying emotions by touch. Past studies defined the relationships between arousal/variance emotion maps and touch characteristics (Russell, 1978) and enabled robots to convey emotions by changing touch characteristics naturally (Meng et al., 2019; Zheng et al., 2020b; Teyssier et al., 2020). Another research focused on conveying intimacy by touch (Zheng et al., 2020a), which will also be useful for caregiver contexts. Related to this topic, researchers have also investigated different locations to be touched based on what emotion is being expressed. One study investigated the relationship between the body locations that were touched and the emotions that were conveyed: e.g., hands/forearms express happiness, and hands/shoulders express sadness (Hertenstein et al., 2006). Another study extended this work with robots and reported that participants mainly touched the hands/forearms of others for both happy/sad emotions with robots (Andreasson et al., 2018; Lowe et al., 2018). This knowledge will help design the touch behaviors of social robots, particularly how robots convey emotions to patients during care. However, human science literature investigated the difference of acceptable touched places due to personal relationships (Suvilehto et al., 2015; Suvilehto et al., 2019). Therefore, social robots should also consider the acceptability of PwDs during touch interaction with them.

Finally, as described in section *Touch Interaction*, touch behavior brings positive or negative meaning to people even if touchers are social robots; moreover, we also need to consider the effects of behavior change effects as well as perceived impressions. Because related works in human-robot touch interaction reported positive effects of robots’ touches toward people’s behaviors, such as improved motivation for monotonous tasks (Shiomi et al., 2017a), persuasion (Bevan and Stanton Fraser, 2015), encouraging self-disclosure and pro-social behaviors (Shiomi et al., 2017b; 2020b), and stress-buffering (Shiomi and Hagita, 2017), similar to human-human touch interactions. Since these studies were conducted with relatively young participants and not PwDs, additional evaluations with PwDs under Humanitude contexts are critical to apply such knowledge in care contexts.

### Technical Challenges for Assistance With Standing up

Many efforts have investigated using robots for assisting seniors to stand and walk on their own, which is a major goal of Humanitude (Martins et al., 2012; Cifuentes and Frizera, 2016; Geravand et al., 2017; Ferrari et al., 2020; Takeda et al., 2020). Robotic technologies do already exist that facilitate the standing or walking of PwDs by themselves. However, few studies exist on systems that assist PwDs with the interaction techniques in Humanitude for producing feelings of comfort and positive impressions of the care. For example, Takeda et al. proposed a sit-to-stand support system that informed users by verbal guidance when assistance will begin. Healthy adult users felt comfortable when the start of the robot’s movement was indicated by voice (Takeda et al., 2020), although they did not evaluate their system with healthy seniors or PwDs. Providing a feeling of comfort through multimodal information in standing and walking is critical for PwDs.

If a robot conveys a feeling of comfort in a multimodal manner, it can provide Humanitude care by collaborating with human caregivers. This allows caregivers to divide interaction techniques with it; the robot is responsible for the skills that are difficult for a caregiver to acquire but easy for the robot to implement. For example, while assisting a PwD to stand or walk, the robot can perform Auto Feedback, which describes what the human caregiver is going to do and will soon be doing while the human caregiver makes eye contact and gently touches the person.

### Technical Challenges for Modeling Care Procedure in Humanitude

To date, no robot can perform all of the stages of care procedure in Humanitude. Therefore, care techniques must be implemented into social robots to cover all stages in addition to achieving such advanced cognitive functions as the recognition of seniors and their environments, and their physical functions to conduct actual care. Humanitude provides appropriate communication strategies at each stage, which could be implemented into a robot. However, people have different impressions of a robot depending on its appearance and size. In fact, a study that presented pictures of various social robots to PwDs concluded that robots that share some traits with humans/animals and machines are more attractive than humanoid robots (Wu et al., 2012). For robots whose appearances are different from humans, their interactions with PwDs may be different from those between PwDs and caregivers. As researchers have done in various HRIs (Kahn et al., 2008; Mutlu and Forlizzi, 2008; Lee et al., 2010), new interaction patterns must be investigated with PwDs based on Humanitude.

We also need to address the development of a system that enables contingent interaction with PwDs since the caregiver’s contingent response is essential, as described in section *Care Procedure to Build Intimate Relationships With Seniors With Dementia*. Such a system also has the potential to give a good impression to users (Yamaoka et al., 2007). Yamazaki et al. analyzed the interaction between caregivers and healthy older people in elderly day-care centers in Japan to develop service robots for elderly care, focusing on verbal and non-verbal behaviors surrounding requests (Yamazaki et al., 2007). Their qualitative analysis suggests that service robots in elderly day-care centers should display availability to multiple visitors simultaneously and respond contingently to individual visitors through non-verbal information such as gaze, head, and body orientation. We must analyze contingent interaction between caregivers and PwDs with quantitative methods to design the robot’s responses. However, we should also consider a possibility that the “effective” modal (verbal, eye contact, facial expression, touch, and laugh) is quite dependent on the individual properties of PwDs. This adaptability and reactive contingency in the care interaction is the deepest part of realizing tender-care communication. But so far, current research on AI and robotics does not reach this stage.

## Discussion

### Possible Caregiving Applications for Social Robots With Humanitude Techniques

In this paper, we focused on Humanitude, an effective comprehensive care technique in elderly care, to create a robot that contributes to reducing BPSDs in PwDs and improves their QoL. As people age, their sensory and cognitive functions generally decline. This is especially evident in PwDs with whom healthy adults often struggle to communicate. Gaze, touch, and speech, which are fundamental behaviors in our communication, are more crucial for PwDs because they often have difficulty in understanding the meaning of linguistic information (McKhann et al., 2011). However, we cannot use the same behaviors as we do on healthy adults. We need to learn appropriate behaviors to communicate smoothly with PwDs. The appropriate behaviors are also helpful for social robots to communicate with PwDs. However, there is a paucity of studies about them in the existing HRI. Humanitude provides necessary care techniques to facilitate smooth communication with PwDs in care and suggests several technical challenges for social robots that interact with them, an idea that has received less attention in robot-assistive elderly care.

Note that we do not aim to implement Humanitude techniques in social robots to replace human caregivers. Rather, we propose developing such robots as a tool to help caregivers build better relationships with PwDs and provide improved care for them. In Humanitude, not only can one person perform all the seeing, talking, touching, and standing assistance tasks; multiple people can share the tasks. Therefore, by introducing at least a part of the Humanitude care techniques into the interaction’s design between robots and PwDs, robots can provide care in collaboration with caregivers. In other words, a robot based on the Humanitude concept will not only help build good relationships with PwDs but also facilitate the division of roles in Humanitude care with caregivers, improving the quality of care provided by caregivers and robots.

The introduction of Humanitude-based robots also provides a new perspective into Humanitude itself. Recent studies in robotics report that PwDs show high acceptance to social robots; robot therapy is another method to reduce BPSDs. One advantage of using social robots is that they can be designed so that their appearance and interaction patterns are attractive to PwDs. Perhaps a robot can be designed that can easily establish good relationships with PwDs. By introducing robots into Humanitude care, we may be able to create new methods in which robots and caregivers work together. Unfortunately, ethical issues emerge when robots are used in elderly care (Feil-Seifer and Matarić, 2011). To protect the dignity of PwDs, these issues must be discussed in actual care situations in cooperation with the caregivers and the families of PwDs (Stahl and Coeckelbergh, 2016).

Based on these considerations, as described in sections *Technical Challenges Toward Artificial Systems That Incorporate Humanitude Techniques*, firstly, social robots will be used as caring teachers for training novice caregivers due to their existing capabilities for caregiving and limited acceptance from PwDs and their families. In fact, some of the described systems in section *Technical Challenges Toward Artificial Systems That Incorporate Humanitude Techniques* would help to analyze the caregiving behaviors of caregivers quantitatively. Due to the advance of social robots’ physical capabilities for caregiving and increasing social acceptance of PwDs and their families toward social robots, the next step is a second caregiver, which supports human caregivers through physical collaborations. Even if such robots did not have enough physical capabilities for caregiving, the robots could work with human caregivers by dealing with conversation partners of PwDs based on knowledge from Humanitude, e.g., eye-contact modeling. Finally, social robots will work as main caregivers by overcoming technical and ethical problems in a future society, but it is still far from current situations. In other words, implementing Humanitude techniques in social robots is still an early stage. Therefore, the developers need to solve technical issues in the beginning. Thus, we discuss what kinds of problems still exist in three interaction skills essential in Humanitude context in the following subsection.

### Issues of Implementing Humanitude Techniques in Social Robots

We revealed that although robot technologies already exist that assist PwDs to stand and walk (the main objective of Humanitude care), many issues must be addressed in three interaction skills. In techniques for face-to-face interaction, we showed that care robots have to autonomously enter the PwD’s field of vision to establish eye contact with them, unlike healthy adults who can spontaneously adjust it. Although Humanitude proposes quantitative indexes, the gaze behavior of trained caregivers must be analyzed and modeled in more detail so that robots and novice caregivers can learn how to successfully make eye contact with PwDs. First-person assessment systems for Humanitude skills (Nakazawa et al., 2020) are another useful approach for this purpose. Such models of eye contact behavior of trained caregivers are expected to be implemented in a robot to achieve smooth interaction with PwDs in future nursing home.

Compared with guidelines for face-to-face interaction, there are many qualitative guidelines in verbal communication and touch interaction. Therefore, we have to quantitatively analyze verbal communication and touch interaction between well-trained caregivers and PwDs to implement such interaction in artificial systems. For example, in verbal communication, we have to investigate how often a robot conducts Auto Feedback during care and what words are appropriate to create positive impressions in PwDs about caregivers and care itself. Multimodal behavior analysis (Ishikawa et al., 2016; Honda et al., 2017) can provide significant insight into these issues. For touch interaction, little quantitative analysis has analyzed how caregivers decide to touch a PwD or how much force should be applied. Modeling touch behavior and verifying the effects of touch interaction are beginning to focus on robots that can perform touch interactions with people. However, since these studies use data measured on healthy adults, data must be collected on touch interactions between PwDs and well-trained caregivers. In fact, such efforts have already begun. For example, Hiramatsu et al. developed a full-body tactile sensor system that is capable of capturing touch information from a skilled caregiver (Hiramatsu et al., 2020). Sumioka et al. developed a system that measured not only such light touches as stroking but also approaching behaviors before being touched (Sumioka et al., 2019). These systems will eventually model the pre-touch and post-touch behaviors of skilled human caregivers and be implemented in robots and caregiver evaluation systems.

The interaction techniques proposed by Humanitude are effective for evoking positive impressions in PwDs about their caregivers and care contents. However, even if these methods are modeled, and robots perform them, and caregivers learn them, they might not necessarily positively affect PwDs. Skilled caregivers perform interaction techniques while confirming that PwDs have positive emotions based on their responses. As mentioned in section *Facial Expression of PwD and Caregivers*, the facial expressions of PwDs are one way to obtain their emotional information. What PwDs say, their reactions to being touched, and such biological signals as their heartbeats and temperature are also useful information. An important challenge is estimating the emotions of PwDs from the multimodal information obtained from their behavior.

## Footnotes

1
https://www.nhs.uk/conditions/dementia/
